# The clinical utility of HTLV-1 viral load measurement

**DOI:** 10.1186/1742-4690-8-S1-A46

**Published:** 2011-06-06

**Authors:** Maria A Demontis, Silva Hilburn, Graham P Taylor

**Affiliations:** 1 Section of Infectious Diseases, Imperial College, London, W2 1PG, UK

## Introduction

High levels of HTLV-1 proviral load in peripheral blood mononuclear cells (PBMCs) have been observed in patients with Adult T-cell Leukaemia Lymphoma (ATLL) and HTLV-1 Associated Myelopathy/Tropical Spastic Paraparesis (HAM/TSP). The proviral load has been reported to fluctuate in both asymptomatic carriers and in symptomatic patients. We aimed to quantify intra-patient variability, to identify “outliers”, and to characterise the range of viral loads in asymptomatic carriers and in patients with HTLV-1-associated diseases.

## Methods

Viral loads were quantified using a SYBR Green-based real-time quantitative polymerase chain reaction, and expressed as a percentage of infected PBMCs.

## Results

1. Mean viral loads were significantly different between asymptomatic carriers and HAM/TSP (p<0.001), and between asymptomatic carriers and ATLL (p=0.001);

2. Viral loads below 1% were found only in asymptomatic carriers;

3. 15% of asymptomatic carriers had a viral load >10%, 3% >20%;

4. The intra-assay CV increases as viral load decreases;

5. The inter-assay CV at low loads is not different from the intra-assay CV;

6. Mean intra-patient CV is 65% (SD 21) in asymptomatic carriers, and 51% (23) in HAM/TSP.

## Discussion

HTLV-1 viral load is highly variable between infected patients. We speculate that asymptomatic carriers with a viral load higher than 10% are more likely to develop HTLV-1 associated diseases. We suggest the use of intra-patient variability (CV) to identify significant variations in viral load, that would allow the identification of patients who may be developing an associated disease, or to assess the efficacy of treatments to reduce viral load.

